# Chimeric Antigen Receptor (CAR) Treg: A Promising Approach to Inducing Immunological Tolerance

**DOI:** 10.3389/fimmu.2018.02359

**Published:** 2018-10-12

**Authors:** Qunfang Zhang, Weihui Lu, Chun-Ling Liang, Yuchao Chen, Huazhen Liu, Feifei Qiu, Zhenhua Dai

**Affiliations:** Section of Immunology and Joint Immunology Program, Guangdong Provincial Academy of Chinese Medical Sciences, and Guangdong Provincial Hospital of Chinese Medicine, Guangzhou, China

**Keywords:** Treg, antigen-specificity, chimeric antigen receptor (CAR), immunological tolerance, transplantation, autoimmunity

## Abstract

Cellular therapies with polyclonal regulatory T-cells (Tregs) in transplantation and autoimmune diseases have been carried out in both animal models and clinical trials. However, The use of large numbers of polyclonal Tregs with unknown antigen specificities has led to unwanted effects, such as systemic immunosuppression, which can be avoided via utilization of antigen-specific Tregs. Antigen-specific Tregs are also more potent in suppression than polyclonal ones. Although antigen-specific Tregs can be induced *in vitro*, these iTregs are usually contaminated with effector T cells during *in vitro* expansion. Fortunately, Tregs can be efficiently engineered with a predetermined antigen-specificity via transfection of viral vectors encoding specific T cell receptors (TCRs) or chimeric antigen receptors (CARs). Compared to Tregs engineered with TCRs (TCR-Tregs), CAR-modified Tregs (CAR-Tregs) engineered in a non-MHC restricted manner have the advantage of widespread applications, especially in transplantation and autoimmunity. CAR-Tregs also are less dependent on IL-2 than are TCR-Tregs. CAR-Tregs are promising given that they maintain stable phenotypes and functions, preferentially migrate to target sites, and exert more potent and specific immunosuppression than do polyclonal Tregs. However, there are some major hurdles that must be overcome before CAR-Tregs can be used in clinic. It is known that treatments with anti-tumor CAR-T cells cause side effects due to cytokine “storm” and neuronal cytotoxicity. It is unclear whether CAR-Tregs would also induce these adverse reactions. Moreover, antibodies specific for self- or allo-antigens must be characterized to construct antigen-specific CAR-Tregs. Selection of antigens targeted by CARs and development of specific antibodies are difficult in some disease models. Finally, CAR-Treg exhaustion may limit their efficacy in immunosuppression. Recently, innovative CAR-Treg therapies in animal models of transplantation and autoimmune diseases have been reported. In this mini-review, we have summarized recent progress of CAR-Tregs and discussed their potential applications for induction of immunological tolerance.

## Introduction

Regulatory T cells (Tregs) are a subpopulation of T cells that can suppress the function of conventional T cells and other immune cells. A major subset of Tregs is defined by their stable expression of the interleukin (IL)-2 receptor α chain (CD25) and the transcription factor forkhead box protein 3 (FoxP3), which determines Treg function (1). It has been shown that mutations in FoxP3 gene impair Treg function, causing severe and lethal autoimmunity, including immune dysregulation, polyendocrinopathy, enteropathy, and X-linked (IPEX) syndrome in mice and humans (2, 3). CD4^+^CD25^+^ Tregs are classified into two subsets: thymus-derived natural Tregs (nTregs) that comprise 5–10% of CD4^+^ T cell compartment and peripherally induced Tregs (iTregs) that develop from naive T cells in the periphery (4–7). Numerous studies have shown that Tregs exert their suppressive function in both contact-independent and contact-dependent manners, including release of inhibitory cytokines, disruption of metabolic pathways, suppression of antigen-presenting cells (APCs), and cytotoxic mechanisms (8–10). Tregs play an important role in preventing graft-vs.-host disease (GVHD) (11), allograft rejection (12), and autoimmune diseases (13–15).

Tregs inhibit allograft rejection and autoimmunity in many animal models (12–18) while multiple Treg-based cell therapies have also been conducted in clinical trials (19). The first clinical trial of adoptive transfer of *in vitro*-expanded Tregs was reported for treating GVHD (20). This cellular therapy allowed for significant alleviation of the symptoms and reduction in conventional immunosuppressive agents in chronic GVHD. Then, multiple clinical trials demonstrated that Tregs prevented both acute and chronic GVHD with no cytotoxicity and other adverse events (21–23). On the other hand, polyclonal Tregs were shown to preserve beta-cell function, prolong pancreatic islet survival and attenuate type 1 diabetes (T1D) (24–26). In these studies, the adoptively transferred Tregs were proved to be safe while ameliorating T1D. As the safety of Treg therapy was demonstrated in these clinical trials, additional trials based on antigen-specific Treg therapies in solid organ transplantation were conducted (10, 27). Using *ex vivo*-generated Tregs, a major pilot study showed that most of the patients developed liver transplant tolerance with normal graft function but with no obvious side effects after the withdrawal of immunosuppressive agents (28). Thus, Treg therapies hold much promise for treating both autoimmune diseases and transplant rejection.

However, previous clinical trials have largely used polyclonal or *in vitro*-expanded Tregs (29). While the initially limited success of polyclonal Tregs is encouraging, the amounts of cells needed for infusions are quite large and the risk of non-specific immunosuppression should be considered. Indeed, viral reactivation after infusion of polyclonal Tregs has been reported (30). These drawbacks could be overcome using antigen-specific Tregs, which require fewer cells to exert more localized and targeted suppression than polyclonal Tregs. Moreover, many groups have demonstrated that Tregs specific for a desired antigen are functionally superior to polyclonal or unmodified Tregs in animal models (27, 31–33).

**Figure 1:**
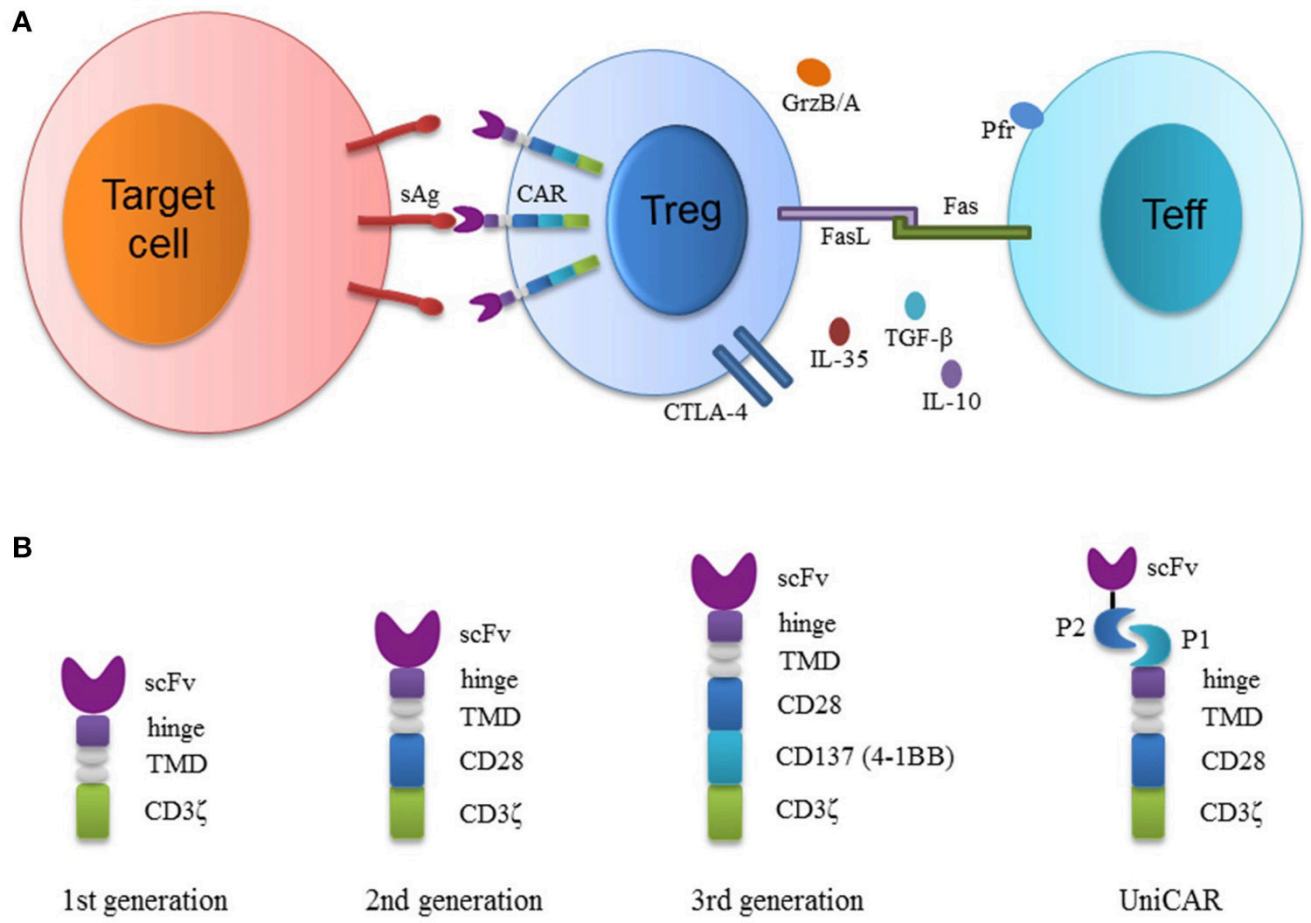
Schematic diagram depicting the structure of CAR-modified regulatory T cells (CAR-Tregs) and their suppression of effector T cells. **(A)** Tregs transduced with viral vectors overexpress CARs that specifically recognize surface antigens on target cells. CAR-Tregs suppress effector T (Teff) cells through various mechanisms. CAR-Tregs secrete immunosuppressive cytokines. CTLA-4 on activated Tregs also competes with CD28 on Teffs to bind CD80/CD86 on APCs. Granzyme B/A (GrzB/A) and perforin (Pfr) secreted by Tregs or their Fas-ligand can induce Teff apoptosis. **(B)** The constructions of the first generation (1st CAR), second generation (2nd CAR), third generation (3rd CAR) and universal CAR (UniCAR) are presented. CARs consist of antigen binding scFv (single chain variable fragment), an extracellular hinge, a transmembrane domain (TMD) and intracellular signaling (CD28/CD137/CD3ζ) domains. The 1st CAR contains only CD3ζ signaling domain. The 2nd CAR contains an additional costimulatory domain (either CD28 or CD137). The 3rd CAR combines both of the costimulatory domains. Finally, the hinge of the universal CAR is attached to P1 (a peptide or protein), which binds to another peptide or protein P2 fused to an scFv recognizing surface molecules on target cells.

Traditional methods to generate antigen-specific Tregs rely on expanding Tregs with APCs and specific antigens or engineering Tregs with T-cell receptors (TCRs). Treg expansion with APCs is inefficient, because there are few antigen-specific Tregs in the original polyclonal cells. Although Tregs engineered with TCRs (TCR-Tregs) seem to be promising (18, 31, 34–36), they are still MHC-restricted, limiting the modular application in individual patients. An MHC-independent strategy of generating antigen specificity is to engineer Tregs with genes encoding chimeric antigen receptors (CARs). CARs typically consist of a single-chain variable fragment (scFv, a binding moiety of monoclonal antibody), an extracellular hinge, a transmembrane region, and intracellular signaling domains (Figure 1) (27). CAR-modified T (CAR-T) cells are now mainly used for cancer immunotherapies. CD19-targeted CAR-T cells are effective in treating hematologic malignancies in preclinical and clinical trials (37, 38), and have been authorized by US FDA for clinical treatments in 2017. This technology has been extended to Treg therapies using Tregs engineered with CARs (CAR-Tregs). In animal models, CAR-Tregs have shown great potential for treating different diseases, especially allograft rejection and various autoimmune diseases. In this mini-review, we have summarized CAR-Treg therapies mainly in transplantation and autoimmunity (Table 1). Also presented is the schematic diagram showing CAR-Treg structure and mechanisms underlying their suppression (Figure 1).

**Table 1:** Potential application of CAR-Tregs for different diseases.

**Disease model**	**Antigen specificity**	**Functional characteristics**	**References**
**TRANSPLANTATION**			
GVHD	HLA-A2	Superior to polyclonal Tregs at preventing xenogeneic GVHD after engraftment with human PBMCs	(39)
Skin Transplant Rejection	HLA-A2	Completely preventing rejection of HLA-A2–positive PBMCs and skin grafts	(40)
Skin Transplant Rejection	HLA-A2	Inhibiting rejection of human HLA-A2–positive skin grafts more effectively than polyclonal Tregs	(41)
GVHD, islet, and Skin Transplantation	Universal	Activation of mAbCAR-Treg by FITC conjugated mAb Preventing GVHD and extending survival of islet allografts and secondary skin allografts	(42)
**AUTOIMMUNE DISEASES**			
Colitis	TNP	Attenuation of murine colitis by CAR-Treg Better than polyclonal Tregs	(43, 44)
Colitis and colorectal cancer	CEA	Better than irrelevant CAR-Tregs in ameliorating colitis and colitis-associated colorectal cancer	(45)
Multiple sclerosis	MOG	Naïve CD4^+^ T cells reprogrammed into Tregs by over-expressing FOXP3 Suppressing EAE better than MOCK-transduced Tregs	(46)
**MISCELLANEOUS**			
Hemophilia A	FVIII	CAR-Treg activated by soluble protein FVIII Suppression of anti-FVIII antibody responses	(47)
Asthma	CEA	More efficient in controlling asthma than unmodified Tregs	(48)
Burkitt lymphoma	CD19	Inhibiting antitumor efficacy of CD19-specific CAR-T	(49)
Sarcoma	CEA	Inhibiting antitumor efficacy of CEA-specific CAR-T	(50)
Prostate cancer	Universal	Activation of UniCAR-Treg by a peptide E5B9 -conjugated mAb/scFv targeting a cell surface structure Costimulation with CD137 superior to that with CD28 in terms of safety issues Inhibiting antitumor efficacy of Teff with the same specificity	(51)

*GVHD, graft-vs.-host disease; HLA, human leukocyte antigen; PBMC, peripheral blood mononuclear cell; mAb, monoclonal antibody; FITC, fluorescein isothiocyanate; TNP, 2,4,6-trinitrophenol; CEA, carcinoembryonic antigen; MOG, myelin oligodendrocyte glycoprotein; EAE, experimental autoimmune encephalomyelitis; FVIII, Factor VIII; scFv, single chain variable fragment*.

## CAR-treg in transplantation

MHC class I molecule is constitutively expressed on the surface of nearly all transplanted cells, including passenger leukocytes emigrating from a graft. In particular, HLA-A2 is highly prevalent (>40%) in white donors (52, 53). In addition, HLA-A mismatching is often associated with poor outcomes after transplantation. Thus, HLA-A2 is a potential target antigen to generate antigen-specific Tregs for inducing transplantation tolerance.

Alloantigen-specific human Tregs were created with a HLA-A2–specific CAR (A2-CAR) in a peptide-independent manner and utilized to prevent xenogeneic GVHD in the immune deficient NOD.SCID.γc-/- (NSG) mice receiving HLA-A2^+^ human PBMCs alone or with A2-CAR-expressing Tregs (39). A2-CAR-Tregs maintained high expression of canonical Treg markers, including FoxP3, CD25, Helios, CTLA-4 and a high degree of demethylation of the Treg-specific demethylated region (TSDR) of the FOXP3 locus. An A2-CAR on Tregs enabled stronger antigen-specific activation than did an endogenous TCR. A2-CAR-Tregs activated via CARs suppressed *in vitro* proliferation of CD8^+^ T cells. Moreover, A2-CAR-Tregs were more efficient in preventing xenogeneic GVHD in the NSG recipients than were TCR-Tregs. Unlike TCRs, CARs could also stimulate IL-2-independent Treg proliferation in short-term (39). Further, CAR-stimulated Tregs had higher surface expression of CTLA-4, latency-associated peptide (LAP) and the inactive precursor of TGF-β. Thus, CAR-Tregs may be superior to TCR-Tregs.

A2-CAR-Tregs were then developed to prevent rejection of skin allograft (40). A2-CAR changed the specificity of nTregs without alteration of their regulatory phenotypes and epigenetic stability. Activation of the Tregs via an A2-CAR led to a stronger cell proliferation and upregulation of CD39 effector molecule, and inhibited allospecific effector T (Teff) cell proliferation *in vitro* more effectively than that of unmodified nTregs or control CAR-Tregs. Furthermore, based on measurements of ear thickness in NOD.Rag1^null^IL-2γc (NRG) mice receiving A2-CAR Tregs or control nTregs plus HLA-A1+ PBMCs (responders) and irradiated HLA-A2+ PBMCs (stimulators), A2-CAR Tregs suppressed allogeneic responses of delayed-type hypersensitivity more potently than did unmodified nTregs or control CAR-Tregs. While adoptive transfer of polyclonal nTregs or control CAR-Tregs had only a moderate effect on tolerance induction, transfer of A2-CAR-Tregs completely prevented killing of allogeneic HLA-A2-positive target cells and rejection of HLA-A2-positive human skin grafts for over 40 days. Histologic examination showed that transferred A2-CAR-Tregs homed to skin grafts and persisted for long-term (40). Meanwhile, a similar study was reported with two HLA-A2-specific CARs engineered: one comprising a CD28-CD3ζ signaling domain (CAR) and another lacking an intracellular signaling domain (ΔCAR) (41). In this model, it was found that, relative to polyclonal Tregs, CAR and ΔCAR Tregs transmigrated through HLA-A2^+^ endothelial monolayers much faster than through HLA-A2^−^ endothelial monolayers *in vitro*, which confirmed a preferential migration of A2-CAR-Tregs into HLA-A2^+^ target tissues (41). This finding suggests that expression of target antigens on transplanted cells would likely stimulate localization of antigen-specific CAR-Tregs to a graft. Indeed, Treg localization to the graft is important to prevent allograft rejection and induce transplant tolerance (54). These two reports on A2-CAR Tregs imply a potentially clinical application for CAR-Treg therapies.

Antonio et al. developed a new type of CAR termed mAbCAR expressing a FITC-targeted CAR on Tregs that could be activated in a flexible way by various mAbs covalently conjugated to FITC (42). They proved that mAbCAR Tregs could be activated by FITC-conjugated antibodies. Antigen-specific mAbCAR Tregs retained their original phenotypes and functions. Prior to receiving allogeneic donor T cells and T cell–depleted bone marrow, adoptive transfer of donor-derived MAdCAM1-mAbCAR Tregs into lethally irradiated allogeneic BALB/c recipients effectively prevented GVHD. Compared with isotype-mAbCAR Tregs, donor-specific H-2D^d^-mAbCAR Tregs, which were directed against an MHC-I antigen H-2D^d^ expressed on transplanted islets, also significantly prolonged islet allograft survival. Bioluminescent imaging and histologic analysis confirmed an enhanced ability of H-2D^d^-mAbCAR Tregs to home to and expand in the islet grafts. They further demonstrated that H-2D^d^-mAbCAR Tregs fostered alloantigen-specific peripheral tolerance by showing that mice infused with H-2D^d^-mAbCAR Tregs exhibited a prolonged survival of secondary skin allografts (42). Thus, the ability to home to target tissues was improved by antigen-specific stimulation of mAbCAR Tregs that could be activated by various FITC-conjugated mAbs recognizing a number of different surface proteins, suggesting that mAbCAR can be used to extend CAR technology to Treg application. Similarly, another flexible module termed universal CAR (UniCAR) has been published recently (51), as described under the subheading of CAR-Treg for Other Diseases.

## CAR-treg in autoimmunity

Elinav et al. initially reported CAR Tregs that were isolated from transgenic BALB/c mice with a CAR specific for 2,4,6-trinitrophenol (TNP), an antigen commonly used in a mouse model of colitis (43). TNP-CAR Tregs, but not non-specific control Tregs, suppressed the proliferation of both control and TNP-CAR Teffs *in vitro*. The costimulatory signal CD28 was not crucial for the Treg suppression as the CAR-Tregs could also inhibit Teff cell proliferation even in the absence of B7-CD28 costimulation. When colitis was induced with 2,4,6-trinitrobenzene sulphonic acid (TNBS), the mortality rate of TNP-CAR-tg mice significantly decreased in comparison with WT mice, indicating that TNP-CAR Tregs may have protected mice from colitis. Similarly, transfer of TNP-CAR Tregs, but not unmodified Tregs, to WT mice with TNBS colitis alleviated the disease and prolonged mouse survival. *In situ* fluorescent micro-endoscopic evaluation verified that TNP-CAR Tregs localized to inflamed colonic mucosa. Moreover, intrarectal administration of TNBS resulted in TNP-CAR Treg-mediated protection from oxazolone-induced colitis, implying that activation of these TNP-CAR Tregs by TNBS also generates “bystander suppression” (43). Thus, Tregs redirected to inflamed tissue can exert protective effects with a specificity that differs from that of pathogenic T cells.

To employ this approach in a non-transgenic mouse model, the same group developed a novel protocol that enabled efficient and reproducible retroviral transduction and expansion of murine nTregs, leading to a highly enriched population of TNP-specific Tregs. The TNP-CAR nTregs exhibited similar suppressive capabilities to TNP-CAR Tregs both *in vitro* and *in vivo*. Furthermore, TNP-CAR nTreg-mediated suppression *in vitro* was partially dependent on cell-cell contact but not IL-10 or TGF-β1 (44). Since carcinoembryonic antigen (CEA) has been shown to be overexpressed in human colitis and colorectal cancer (55), this group also utilized CEA-specific CAR-Tregs to treat CEA-transgenic mice with colitis or colorectal cancer. Two experimental models were implemented: CEA-specific CAR Teff-induced colitis and azoxymethane–dextran sodium sulfate (AOM–DSS) murine model of colitis-associated colorectal cancer. CEA-CAR Tregs suppressed the proliferation of CEA-CAR Teffs more effectively than did the irrelevant control CAR Tregs *in vitro*. Both CEA-CAR Tregs and CEA-CAR Teffs, but not control CAR Tregs, homed to and accumulated in the colon of diseased mice, confirming the importance of antigen-specific stimulation for Teff or Treg homing to target organs. On the other hand, transfer of CEA-CAR Tregs ameliorated CEA-CAR Teff-transferred colitis, AOM–DSS-induced colitis and colitis-associated colorectal cancer more efficiently than that of control CAR Tregs (45).

In another study, CAR Tregs were engineered with specificity for myelin oligodendrocyte glycoprotein (MOG) to suppress experimental autoimmune encephalomyelitis (EAE), a model relating to multiple sclerosis in humans (46). In this model, murine FoxP3 was co-expressed with the CAR to drive Treg differentiation from naïve CD4^+^ T cells. Reprogramming naïve or memory CD4^+^ T cells into Tregs by overexpressing a high level of FOXP3 has been proved to be valid (56, 57). In these studies, engineered T cells obtained Treg suppressive capacity. MOG-CAR Tregs efficiently homed to various regions in the brain after intranasal cell delivery, as demonstrated by examination of horizontal cryosections of the brain, and suppressed ongoing encephalomyelitis, as evidenced by reduced disease symptoms and decreased mRNA levels of IL-12 and IFN-γ in the brain tissue. Moreover, EAE mice treated with MOG-CAR Tregs were protected from a second EAE challenge, indicating a sustained effect of the engineered CAR-Tregs (46).

## CAR-treg for other diseases

Hombach et al. pioneered CEA-specific CAR-Tregs in 2009 (50). Although cytolytic immune responses of CEA-specific CD3^+^ T cells were dramatically repressed in the presence of CEA-CAR Tregs, the low expression of FOXP3 and coproduction of IFN-γ and IL-10 make it difficult to assess the net impacts of CAR expression on Treg suppression. Since CEA is mainly present on the surface of adenoepithelia in the lung and gastrointestinal tract, Skuljec et al. redirected CEA-CAR Tregs toward lung epithelia in a mouse model of experimental allergic asthma (48). CEA-CAR Tregs significantly reduced *in vitro* proliferation of CEA-CAR Teffs stimulated through their CARs. After adoptively transferred to asthmatic CEA transgenic mice, CEA-CAR Tregs were activated and accumulated in the lung and tracheobronchial lymph nodes, reduced airway hyperreactivity and diminished eosinophilic airway inflammation more efficiently than unmodified Tregs (48). In mice lacking transgenic CEA, however, CEA-CAR Tregs functioned similarly to unmodified Tregs in suppression of inflammation and were not accumulated in the lung, supporting the view that antigen-specific stimulation enhances the ability of Tregs to home to target sites and exert suppression.

Based on a previous study showing that CD19-CAR T cells successfully eradicated systemic human CD19^+^ tumors in SCID-Beige mice (58), Lee et al. engineered CD19-targeted CAR Tregs (49). These Tregs exerted immunosuppressive function and facilitated a hostile tumor microenvironment, in which antitumor activity of CD19-CAR Teffs was suppressed. When tumor-bearing SCID mice transferred with CD19-CAR Tregs were lymphodepleted with cyclophosphamide, the subsequently infused CD19-CAR Teffs restored their antitumor capacity, suggesting that antigen-specific CAR-Tregs hinder antitumor activity of CAR-T cells.

FVIII-specific chimeric antigen receptor (ANS8-CAR) Tregs were engineered to generate tolerance to factor VIII (FVIII) in a model of hemophilia A (47, 59, 60). Soluble FVIII, not a protein on the cell surface, could be used as a target antigen for antigen-specific Treg recognition. When activated by FVIII, ANS8-CAR Tregs suppressed anti-FVIII antibody responses. These results were consistent with important data using FVIII-specific TCR-Tregs published by the same group (31). Interestingly, ANS8-CAR Tregs specific for A2 domain of FVIII suppressed *in vitro* proliferation of effector T cells with a specificity toward a C2 domain of FVIII or a different protein, myelin basic protein (MBP) (47), suggesting that CAR-Tregs can exert “bystander” suppression *in vitro* in the presence of both antigens that activate Tregs and T effectors. It remains to be determined if this bystander suppression occurs *in vivo*. It is also unclear whether this bystander immunosuppression generated by antigen-specific CAR-Tregs is superior to deleterious non-specific immunosuppression by polyclonal Tregs.

Universal CAR-Tregs (UniCAR-Tregs) have been recently developed from tumor-targeted CAR-Ts (51). The activation of UniCAR-Tregs is mediated by a peptide epitope-conjugated scFv, which targets a cell surface structure. UniCAR-Tregs can be applied universally as their antigen-specificity is easily adjusted using scFvs with different specificities. UniCAR-Tregs have demonstrated greater suppressive capacity than did unmodified Tregs. Moreover, UniCAR-Tregs with CD137 co-stimulation molecule showed a stronger suppressive activity *in vivo* than did UniCAR-Stop Tregs transduced with intracellular signaling domain-deficient UniCAR (51).

## Advantages and limitations of CAR-tregs

It is generally accepted that antigen-specific Tregs, including TCR- and CAR-Tregs, are superior to polyclonal Tregs in their suppression. Tregs need to migrate to disease-related target organs to exert maximal effects of suppression. Thus, CAR-Tregs are more potent in suppression than polyclonal Tregs since antigen-specific CAR-Tregs tend to migrate to a target organ harboring a specific antigen. Compared with non-specific immunosuppression mediated by polyclonal Tregs, inhibition mediated by CAR-Tregs is antigen-specific, which likely generates fewer side effects related to general immunosuppression. They provide an approach to achieving disease-specific immunosuppression. Moreover, CAR-Tregs appear to hold advantages over TCR-Tregs given that the former is non-MHC-restricted and less dependent on IL-2 than the latter.

However, there are some disadvantages or limitations in CAR-Tregs. It is well known that treatments with anti-tumor CAR-T cells can cause side effects related to cytokine “storm” and neuronal cytotoxicity. It remains to be determined whether CAR-Tregs would also induce these adverse reactions. Furthermore, there is a need to characterize antibodies specific for self- or allo-antigens to construct efficient and specific CAR-Tregs. Selection of antigens targeted by CARs and development of specific antibodies are time-consuming and may be difficult in some disease models. Finally, exhaustion of CAR-Tregs likely limits their efficacy in suppression. Because CD28 plays an important role in Treg development and expansion (61), most of the studies on CAR-Tregs used the second generation CARs with CD28 costimulatory domain to expand the Tregs while some studies indicated that CARs with CD137 costimulation, but not CD28, could ameliorate T cell exhaustion, thus improving CAR-T persistence (62, 63). Perhaps, inclusion of both CD28 and CD137 costimulatory domains may further help maintain CAR-Tregs. More studies are warranted to select the best costimulatory signals for optimal CAR-Treg suppression. Further, CARs incorporating IL-2 receptorβ-chain (64), telomerase reverse transcriptase co-transduction (65) or treatment with PI3K inhibitor (66) were found to improve *in vivo* persistence of CAR-T cells. It remains to be defined whether these measures would also improve CAR-Treg performance.

## Perspective

With the development of the new generation of CAR-Ts, such as UniCAR-T (51, 67), and genome-editing technologies to eliminate the immunogenicity of endogenous TCRs and MHC molecules (68, 69), donor-derived or third-party T cells may be used to modularly generate CAR-T or CAR-Treg cell banks with batch production and improved safety. In contrast, autologous CAR-Tregs made individually are time-consuming although they are not immunogenic. A recent study has reported that genome-editing can be utilized to identify neoantigens for cancer immunotherapy (70). This technology may also help identify hidden self-antigens. Thus, the development of self-antigen-specific antibodies for CAR construction is expected to accelerate. As researchers make progress in CAR-Treg treatments in animal models, clinical trials using CAR-Tregs will emerge in the near future.

## Author contributions

QZ and WL wrote the manuscript. CL-L, YC and HL collected the literature and provided general idea. FQ and ZD edited the manuscript.

### Conflict of interest statement

The authors declare that the research was conducted in the absence of any commercial or financial relationships that could be construed as a potential conflict of interest
